# The Early Phase of β2m Aggregation: An Integrative Computational Study Framed on the D76N Mutant and the ΔN6 Variant

**DOI:** 10.3390/biom9080366

**Published:** 2019-08-14

**Authors:** Rui J. S. Loureiro, Diogo Vila-Viçosa, Miguel Machuqueiro, Eugene I. Shakhnovich, Patrícia F. N. Faísca

**Affiliations:** 1 BioISI—Biosystems & Integrative Sciences Institute, Faculdade de Ciências, Universidade de Lisboa, Campo Grande, 1749-016 Lisboa, Portugal; 2 BioISI—Biosystems & Integrative Sciences Institute and Departamento de Química e Bioquímica, Faculdade de Ciências, Universidade de Lisboa, 1749-016 Lisboa, Portugal; 3 Department of Chemistry and Chemical Biology, Harvard University, Cambridge, MA 02138, USA; 4 BioISI—Biosystems & Integrative Sciences Institute and Departamento de Física, Faculdade de Ciências, Universidade de Lisboa, 1749-016 Lisboa, Portugal

**Keywords:** protein folding, intermediate states, protein aggregation, protein–protein docking, molecular dynamics, Monte Carlo

## Abstract

Human β2-microglobulin (b2m) protein is classically associated with dialysis-related amyloidosis (DRA). Recently, the single point mutant D76N was identified as the causative agent of a hereditary systemic amyloidosis affecting visceral organs. To get insight into the early stage of the β2m aggregation mechanism, we used molecular simulations to perform an in depth comparative analysis of the dimerization phase of the D76N mutant and the ΔN6 variant, a cleaved form lacking the first six N-terminal residues, which is a major component of ex vivo amyloid plaques from DRA patients. We also provide first glimpses into the tetramerization phase of D76N at physiological pH. Results from extensive protein–protein docking simulations predict an essential role of the C- and N-terminal regions (both variants), as well as of the BC-loop (ΔN6 variant), DE-loop (both variants) and EF-loop (D76N mutant) in dimerization. The terminal regions are more relevant under acidic conditions while the BC-, DE- and EF-loops gain importance at physiological pH. Our results recapitulate experimental evidence according to which Tyr10 (A-strand), Phe30 and His31 (BC-loop), Trp60 and Phe62 (DE-loop) and Arg97 (C-terminus) act as dimerization hot-spots, and further predict the occurrence of novel residues with the ability to nucleate dimerization, namely Lys-75 (EF-loop) and Trp-95 (C-terminus). We propose that D76N tetramerization is mainly driven by the self-association of dimers via the N-terminus and DE-loop, and identify Arg3 (N-terminus), Tyr10, Phe56 (D-strand) and Trp60 as potential tetramerization hot-spots.

## 1. Introduction

Protein aggregation is a process whereby monomeric proteins self-associate to form higher-order oligomers (i.e., dimers, trimers, tetramers, etc.). Often, the final products of protein aggregation are amyloids, i.e., elongated unbranched fibrils consisting of β-structures of separate monomers positioned perpendicular to the fibril axis and stacked strictly above each other [1].

Since amyloids are often associated with disease (e.g., Parkinson’s, Alzheimer’s, and several systemic amyloidoses [2]), establishing the mechanisms of protein aggregation leading to amyloids is particularly important. This task, however, is proving more challenging than solving the folding puzzle. Interestingly, evidence accumulated during the last decade has highlighted the fact that the folding and aggregation mechanisms can be directly connected via monomeric states with aggregation potential that are en-route to the native structure. A paradigmatic example of a protein populating such an intermediate state is human beta-2-microglobulin (b2m), the causing agent of dialysis related amyloidosis (DRA), a systemic disorder that affects the osteoarticular system of patients undergoing long-term (>10 years) hemodialysis.

Studying the aggregation mechanism of b2m in vitro under physiological conditions is particularly challenging (e.g., a concentration of b2m higher than that present in the serum of DRA patients (i.e., 100–200 μM) is not enough to trigger aggregation [3]). Despite this, in vitro studies have shown that under physiological conditions, b2m populates an intermediate state (termed I_T_) that is structurally characterized for having a non-native trans isomerization of Pro32 while retaining the native fold. It is widely accepted that the isomerization of the histidyl–prolyl bond is an important event in b2m fibrillogenesis, but a key role for I_T_ in that process lacks consensus (reviewed in [3]).

In order to study b2m aggregation in vitro under physiological conditions, researchers have been focusing on engineered [4,5,6,7,8,9,10,11,12,13,14,15,16,17,18,19] or natural variants of b2m that self-associate. The most frequently studied natural variants are the cleaved variant ΔK58 [20,21,22,23,24], and ΔN6 [20,25,26,27,28,29,30], which lacks the six N-terminal residues. ΔN6 constitutes ~30% of the ex vivo fibrils of DRA patients [31] and displays a high tendency to aggregate in vitro, forming fibrils even at neutral pH [28]. Moreover, its aggregation potential is enhanced by a mild acidification of the medium (pH 6.2), such as that occurring in the synovial fluid of DRA patients [29]. Interest in ΔN6 has increased substantially in the follow-up of a proposal by Radford and co-workers, according to which it conformationally converts wild-type (wt) b2m into an amyloidogenic state in a mechanism akin to prion conversion [29]. This ‘prionlike hypothesis’ was severely criticized and challenged by Bellotti and co-workers, who reported that wt b2m does not fibrillate with monomeric ΔN6 but rather with preassembled fibrils of ΔN6 [32]. Recently, Estácio et al. [25] used an integrated computational approach to propose that the deletion of the N-terminal hexapeptide triggers the formation of a nativelike intermediate state for folding and aggregation that shares with I_T_ the non-native trans isomerization of Pro32 and a well-preserved core, but it also exhibits an unstructured strand A. The latter becomes maximally detached from the core at pH 6.2, resulting in a higher potential to self-associate [25]. This theoretical prediction provides a mechanistic rationale for the higher aggregation potential at the pH of inflamed joints.

Despite interest in the ΔN6 variant, its biological significance is not clear. Indeed, since ΔN6 has not been found in the circulation of DRA patients, one cannot exclude the possibility that the proteolytic cleavage of the hexapeptide only occurs after fibril assembly, which would invalidate the use of monomeric ΔN6 as a model system to gain insights into b2m aggregation.

More recently, the naturally occurring mutant D76N has come into the spotlight as a biologically and clinically motivated model to study b2m aggregation. D76N is the causing agent of a fatal hereditary systemic amyloidosis affecting visceral organs that was found in one French family. In contrast with the wt form, the D76N mutant readily aggregates in vitro at physiological unseeded conditions, even at low concentrations. It exhibits a considerably lower thermal stability and abundantly populates (~25%) an intermediate state which is structurally compatible with I_T_ [32]. According to results from classical molecular dynamics simulations [4], the D76N I_T_ intermediate displays an enhanced β-sheet forming propensity in the disordered D-strand, an increased exposure of hydrophobic residues to the solvent, and a solvation free energy higher than that of the I_T_ intermediate populated by the wt form. Recently, we explored the folding space of D76N with discrete molecular dynamics simulations of a full atomistic Go potential [33]. Our study predicts that the mutant populates two intermediate states with aggregation potential termed I_1_ and I_2_. They share a well-preserved core but show significant differences in the terminal regions. In particular, I_2_, which is the most aggregation-prone species, is exclusively populated by D76N and features two unstructured termini, while I_1_, which is also populated by the wt form, features the C-terminus unstructured [33]. In a posterior study, Le Marchand and co-workers reported the existence of an intermediate state of D76N that is topologically compatible with I_2_. Indeed, by combining in crystallo with in silico data, the authors reported that D76N sparsely populates a highly dynamic conformation that exposes very aggregation prone regions as a result of a loss of β-structure at the N- and C-terminal strands. Conformational dynamics in this species are especially enhanced relative to the wt protein in the EF-loop and residues located at the end of strand A. The authors associated the higher aggregation propensity of D76N to the destabilization of its outer strands, in line with predictions from simulations [34].

The identification of monomeric species with the potential to self-associate is the first step towards establishing the mechanism of aggregation. The latter implies the identification of all microscopic steps leading to mature fibrils, the rate constants governing each step, and determining the manner according to which they depend on protein sequence and environmental conditions (e.g., pH). Ideally, it should be possible to determine the size distribution and structures of the oligomeric assemblies, filaments, protofibrils and fibrils that form along the amyloid cascade [35]. In the case of b2m, there is experimental evidence supporting the view that the dimerization of aggregation prone monomers is the first step of fibrillogenesis under physiological conditions [36,37,38,39] and that the aggregation pathway of wt b2m proceeds exclusively by the formation of even-numbered oligomers (soluble tetramers and hexamers) formed through the addition of dimeric units [40,41].

In the present study, we conducted an extensive comparative analysis of the dimerization process of D76N and ΔN6 through rigid-body docking simulations of monomeric conformations representative of I (ΔN6), I_1_ (D76N) and I_2_ (D76N) populated under acidic and physiological conditions. In the case of D76N, whose folding space embeds two intermediate states, we analyze the interfacial regions of both homo and heterodimers. We also report first glimpses into the interfacial structure of D76N tetramers resulting from the association of dimers of I_2_ at physiological pH. To gain mechanistic insights into Radford’s ‘prionlike hypothesis,’ we investigated the formation of heterodimers resulting from the association of monomeric conformations representative of the native state of wt b2m and the native state of ΔN6 under relevant pH conditions.

The cost function used in the docking simulations optimizes the interfaces for shape, hydrophobic and electrostatic complementarity; a specific term for intermolecular hydrogen bonds is also included. The novel cost function is an improved version of that used in previous studies [25,33], which optimizes dimer interface for shape complementarity by only taking into account packing interactions.

The deployed simulation approach delivers a structurally resolved picture of the dimerization and tetramerization interface that recapitulates previous experimental findings, and provides novel predictions regarding the structural regions and residues that trigger aggregation as a function of pH.

## 2. Materials and Methods

Previously, we explored the dimerization phase of ΔN6 [25] and D76N [33] as a function of pH by means of a three-stage methodological approach that comprises discrete molecular dynamics (DMD) simulations, constant pH molecular dynamics (CpHMD), and rigid-body Monte Carlo docking simulations based on a cost function that only takes into account packing interactions to optimize shape complementarity. In what follows, we provide an overview of our approach and describe with more detail a novel version of the Monte Carlo ensemble docking (MC-ED) protocol that was employed to perform the comparative analysis reported here. The reader is referred to our previous publications for details and results on DMD and CpHMD molecular dynamics simulations [7,25,33,42].

### 2.1. Overview of the Methodological Approach

We start by saying that the adopted methodological approach does not consider the possibility of protein dimerization occurring concurrently and concomitantly with folding, a situation that could lead to domain-swapped dimers [30]. Instead, it considers the scenario according to which protein–protein association occurs upon the formation of intermediate states en route to the native state, which have the potential to trigger the aggregation pathway. Such folding intermediates must be aggregation prone (by exposing hydrophobic patches) and thermodynamically stable enough (i.e., sufficiently long-lived) to allow for the establishment of intermolecular interactions. Therefore, the first step in our approach was the exploration of the equilibrium folding space of the considered model systems and the identification of aggregation-prone intermediate states. This was achieved via replica-exchange DMD simulations of a full atomistic protein representation combined with a simple, structure-based Go potential. Simple Go potentials are native centric, which means that folding energetics is exclusively driven by native interactions. Since Go potentials do not incorporate non-native interactions, they will not be able to capture misfolding processes leading to compact non-native states or, more generally, regions of the folding free energy landscape where non-native interactions may play a determinant role (e.g., the denatured state). The aggregation-prone intermediates we identified previously [25,33] were native-like in the sense that they exhibit a well-preserved native core but feature unstructured termini.

While DMD simulations allow capturing the timescale of protein folding, they do not incorporate the pH, which is known to be an important environmental parameter in protein aggregation. Indeed, the pH modulates the charge of the ionizable side-chains, which can induce large-scale conformational changes (e.g., the modification of secondary/tertiary structure content [43]) and, more often, minor structural modifications that may have a direct impact on protein–protein association. Furthermore, the modulation of the ionizable side-chains controls the pattern of intermolecular electrostatic interactions established upon protein–protein association. To assess the effect of pH on the dimerization process of b2m we conducted CpHMD with explicit titration starting from conformations representative of the structure-based folding intermediates. This approach generates ensembles of conformations representative of intermediate states at a specific pH (i.e., whose structure and charge has been modulated by the pH), which are subsequently used to study dimerization via protein–protein docking. The outcome of docking simulations was an ensemble (1000–1500 conformations) of (homo or hetero) dimers formed by monomers of intermediates under different pH conditions (e.g., an ensemble of dimers of I_1_–I_1_ intermediates at acidic pH, an ensemble of dimers of I_1_–I_2_ intermediates at physiologic pH, etc.). The dimers within each ensemble were then subjected to a classical molecular dynamics protocol of structure relaxation to remove clashes and other structural errors. The resulting dimers of I_2_ were subsequently docked in order to produce an ensemble of (1000) tetramers. The relaxed ensembles of dimers were eventually structurally analyzed to get information about the triggers of aggregation, i.e., the most likely regions initiating the process, and, at a finer level, the residues that will most likely establish a larger number of intermolecular interactions, acting as aggregation hot-spots.

### 2.2. Monte Carlo Ensemble Docking

The Monte Carlo ensemble docking (MC-ED) was originally introduced in Reference [44] to study the dimerization phase of the small folding domain spc-SH3. It is a low-resolution rigid-body docking procedure that creates protein complexes with an interface optimized for shape complementarity by using a cost function that uniquely considers packing interactions.

**Figure 1:**
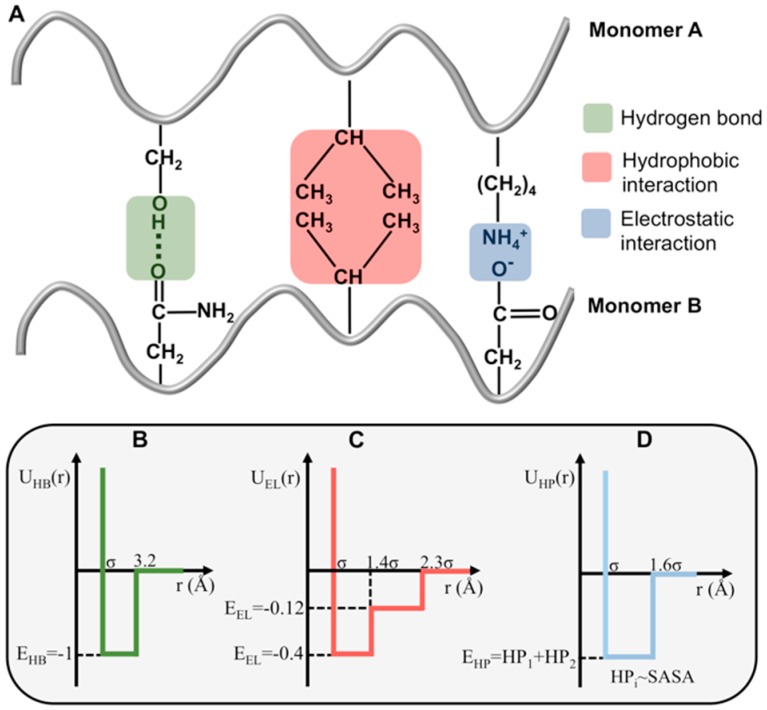
Intermolecular interactions involved in dimerization. Dimer formation is driven by electrostatic interactions (including hydrogen bonds) and interactions between hydrophobic residues (**A**), which are modeled by one or two-step potentials (**B**–**D**).

While shape complementarity is considered one of the main drivers of protein–protein association, it is known that electrostatic interactions (including hydrogen bonds) and hydrophobicity (Figure 1A) also play an important role [45,46,47]. Therefore, in this study, we considered a more realistic cost function that optimizes the complex’s interface for shape, electrostatic and hydrophobic complementarity, the so-called triad of protein–protein association. The protein representation used in the docking simulations considers all atoms (including hydrogen) explicitly. We followed Urbanc et al. [48,49] and used square-well potentials to model inter-atomic interactions (Figure 1B–D). In what follows, we describe the three types of considered intermolecular interactions.

Hydrogen bonds (h-bond) occur when a donor (D) atom donates its covalently bonded hydrogen atom to an electronegative acceptor (A) atom, D–H···A (D, A = N, O, S). In a protein–protein association, an h-bond can be established between the backbones of two interacting chains (i.e., with the donor atom located at one of the backbones and the acceptor atom located at the other), or between pairs of (acceptor–donor) atoms pertaining to the side-chains located at the interfaces of the resulting protein complex. In this study, the distance between donor and acceptor atom was the only criteria to determine the occurrence of intermolecular h-bonds. The reasons are threefold: (1) There is no consensus regarding the geometric constraints that should be imposed when modelling hydrogen bonds [50,51,52]; (2) the linearity of the hydrogen bond is not a stringent requirement for its establishment because hydrogen bonds can be found in other geometric arrangements (which are, nevertheless, weaker) [53]; and (3) to keep the method as computationally efficient as possible. In order to model h-bond interactions, we considered a square-well potential energy function (Figure 1B), whose width ranges between the hard-core distance σ (which is the sum of the van der Waals radii [54] of the two interacting atoms) and a cut-off of 3.2 Å, which is considered the maximum distance for the establishment of a moderately stable (1–5 kcal/mol) hydrogen bond [55,56,57,58]. Since hydrogen bonds in protein–protein interfaces are usually geometrically less optimal (and therefore less stable) than those in the protein interior [53], we took a conservative choice for the corresponding interaction strength $E_{HB}=3$ kcal/mol. This approach also avoids overestimating the contribution of non-geometrically optimal h-bonds for dimer stability. As in Urbanc et al. [55], we set the potential energy for the h-bond to unit energy ($E_{HB}=-1$).

Following References [48,55], we approximated the electrostatic interaction between two charged atoms by a double square-well potential. Two atoms with charges of the same sign interact through a positive (i.e., repulsive) two-step potential, while the interaction between two atoms with opposite charge is modeled through a negative (i.e., attractive) two-step potential (Figure 1C). For these pairwise interactions the signs of the atomic charges in the GROMOS 54A7 force field were used [59]. The protonation states of the protein’s titrable groups at each pH were assigned accordingly to their p*K*_a_ values, obtained from the CpHMD simulations. The cut-off distance was set to 2.3σ, and the width of first potential well is σ < *r* < 1.4σ (short-range interactions 4–5 Å) [60]. The interaction strength corresponding to the first potential well,$E_{EL1}$, was obtained by normalizing the median free energy gain upon salt bridge formation at the protein surface (1.2 kcal/mol) [55,61,62] to the interaction strength of an hydrogen bond (i.e., 3.0 kcal/mol in our model). As in [48], we set the interaction strength of the second potential to $E_{EL2}=0.3\times E_{EL1}$.

To include amino acid interactions that result from the amino acid’s hydropathic nature, we considered a third square-well potential that captures interactions between pairs of hydrophobic/hydrophilic atoms within the side-chains (Figure 1D). The interaction energy was negative (positive) when the distance between two hydrophobic (hydrophilic) atoms was smaller than 160% of the sum of their van der Waals radii [63], which was the cut-off distance below which the interfacial volume was considered as solvent-excluded.

The potential energy between two interacting atoms is defined as $E_{HP}=HP_{1}+HP_{2}$, with $HP_{t}=-s_{t}\times SASA_{t}÷n_{t}$ being the hydropathy value of a specific type of atom *t*. In this equation, $s_{t}$ is the atomic solvation parameter of atom *t* (which corresponds to the free energy gain/loss per unit of solvent-exposed area), $SASA_{t}$ is the solvent accessible surface area of atom *t*, and $n_{t}$ is an estimate for the number of neighboring atoms (usually two). We used the values of the atomic solvation parameters derived by Cummings et al. [64] (Table 1), based on the transfer free energies obtained by Fauchère et al. [65], using the solvent accessible surface areas reported by Lesser et al. [66]. These parameters have a higher discriminating power in the evaluation of protein–protein docking solutions [64]. The hydropathy value of each interacting atom was normalized to the energy of a hydrogen bond, which results in hydrophatic parameters within the interval $0.1\leq HP_{t}\leq 0.4$.

In the MC-ED protocol, the center-of-mass of one of the monomers (or dimers) was kept fixed at the origin of a Cartesian coordinate axis system, and at each MC step the other monomer (or dimer) was allowed to move (i.e., to perform a random rotation or random translation) along the docking axis. The total energy of the complex’s interface contained the three contributions discussed above, i.e.,

(1) $$U=\sum_{i,j}U_{ij}^{H}+U_{ij}^{EL}+U_{ij}^{HP}$$

with $U_{ij}^{H}=U_{H}(r)$ (Figure 1B), $U_{ij}^{EL}=U_{EL}(r)$ (Figure 1C), and $U_{ij}^{HP}=U_{HP}(r)$ (Figure 1D).

At the end of each MC run, a dimer (or tetramer) was generated whose interface was optimized to have a minimum number of clashes and a minimal interfacial energy. The MC-ED was consecutively applied to randomly selected pairs of monomers (or dimers) until the mean and standard deviation of the binding energy both converged. At that point, an ensemble of dimers (or tetramers) containing 1000–1500 (or 1000) conformations was generated. Further information and details on the operational implementation of the MC-ED procedure can be found elsewhere [25,44].

The conformations within each dimer ensemble were finally subjected to a classical protocol of structure relaxation to ensure GROMOS 54A7 compatibility. This protocol started with three energy minimizations steps followed by three restrained MD steps. For the minimization, the first two steps (steepest descent and conjugated gradient) were performed without constraints followed by a steepest descent step with all bonds constrained by the p-LINCS algorithm. The MD steps were performed for 100 ps (the first) and 200 ps (the last two) with gradually decreasing position restraints (1000–10 kJ nm^−2^ mol^−1^). The first were performed at NVT using Berendsen thermostat (310 K with a coupling constant of 0.01) while, in the other two, the pressure was also held constant using Berendsen barostat at 1 atm (with a coupling constant of 2.0 and an isothermal compressibility of 4.5 × 10^–5^ bar^−1^). The GROMOS 54A7 force field was used to perform the relaxation protocol with GROMACS 4.0.7. All GROMOS typical parameters were used namely: 2 fs time step, a twin-range cut-off (8 Å and 14 Å), a reaction-field with a dielectric constant of 54.0 to treat electrostatic contributions beyond 14 Å, and the dimers were solvated with SPC water molecules ensuring that one monomer only interacted with the other in one direction (15,000–35,000 water molecules). About 75% of the dimer structures produced with the MC-ED docking protocol were successfully relaxed to the classical force field. The other 25% were discarded because they had significant conformational clashes that lead to very high-energy interactions that the minimization algorithms were not able to correct. The adopted protocol drove a local structure relaxation of protein complexes, which means that the oligomerization interface never changed significantly after the relaxation step. At this point, we feel that a word of caution is necessary. Since the MC-ED is a rigid-body procedure and the relaxation step only allows for local structure relaxation, the adopted methodology is not able to capture large structural changes that may accompany protein association; therefore it cannot be used to make accurate predictions on oligomer structure. However, this limitation should not be determinant in the context of the present work, whose ultimate goal was to predict the residues that trigger protein–protein association establish by establishing a higher number of intermolecular interactions. Indeed, the dimerization (and, by extension, aggregation) hot-spots should depend, in the first place, on the encounters between monomers (and on how monomers face each other. The ensemble nature of the docking method naturally incorporates this dependence by considering a significantly large number of monomer pairs with different relative orientations. The method’s limitation to capture large conformational changes may be more relevant to identify the hot-spot residues that trigger tetramerization. However, by also capturing the structural heterogeneity of protein–protein association at the tetramer level, the method mitigates—at least up to a certain extent—the limitation resulting from its rigid-body nature.

## 3. Results and Discussion

### 3.1. Model Systems

**Figure 2:**
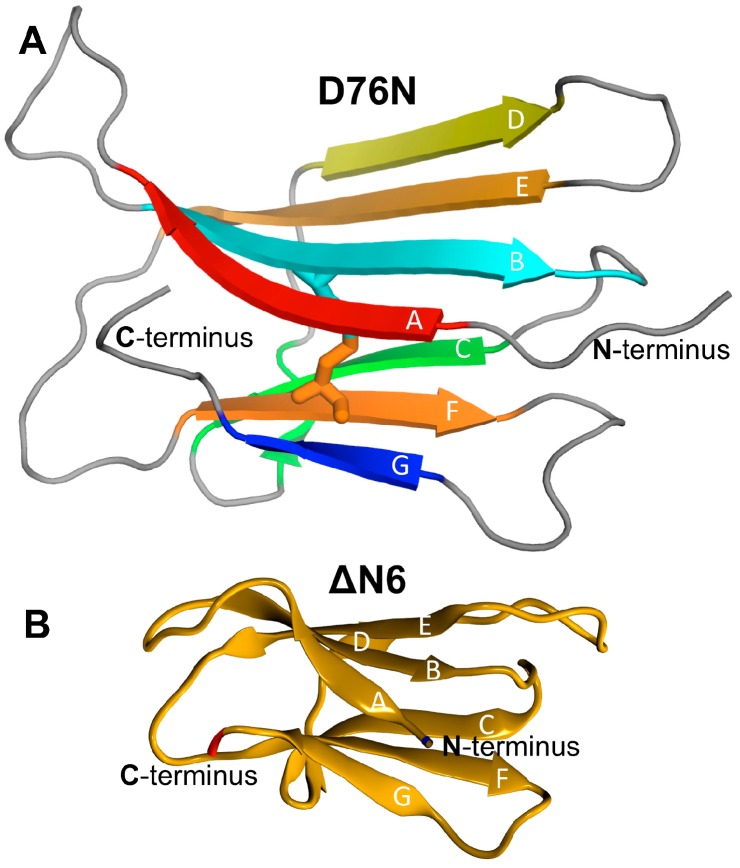
Model systems. (**A**) Native structure of the D76N mutant of protein beta-2-microglobulin (PDB ID: 4FXL), highlighting the seven beta-strands and the disulfide bond between Cys25 and Cys80; (**B**) native structure of the ΔN6 variant (PDB ID: 2XKU), which lacks the first six N-terminal residues.

b2m is a 99 amino acid long chain that folds natively into a seven-stranded antiparallel β-sandwich formed by two sheets of antiparallel β-strands (Figure 2). One of the sheets comprises strands A–B–E–D, and the other sheet contains strands C–F–G. The two β-sheets are linked covalently by one intramolecular disulfide bond between Cys25 (located on strand B) and Cys80 (located on strand F), which is required for aggregation in vitro [67]. Another key structural feature of b2m is the existence of a peptidyl–prolyl bond on the BC-loop (between His31 and Pro32), which adopts a thermodynamically unfavorable cis isomerization in the native state.

With the exception of a higher isoelectric point and a new hydrogen bond between Asn76 and Tyr78, the native structure of the D76N mutant (Figure 2A) matches closely that of the wt form [68].

ΔN6 is a truncated variant of b2m, which lacks the first six N-terminal residues (Figure 2B). Radford and co-workers proposed that ΔN6 is a structural mimic of the intermediate I_T_ because it populates a conformational state that reproduces the conformational features of I_T_ and represents 90% of ΔN6’s in vitro equilibrium population [37]. In line with this finding, ΔN6 reveals a major repacking of the hydrophobic core to accommodate the nonnative peptidyl–prolyl trans-isomer at Pro32.

In the present study, we used the high-resolution crystal structure of Goto and co-workers as a model system for the wt form under physiological conditions (PDB ID: 2YXF) and that of Bellotti and co-workers as a model system for the D76N mutant (PDB ID: 4FXL). For ΔN6, we used the NMR structure determined by Radford and co-workers (PDB ID: 2XKU).

**Figure 3:**
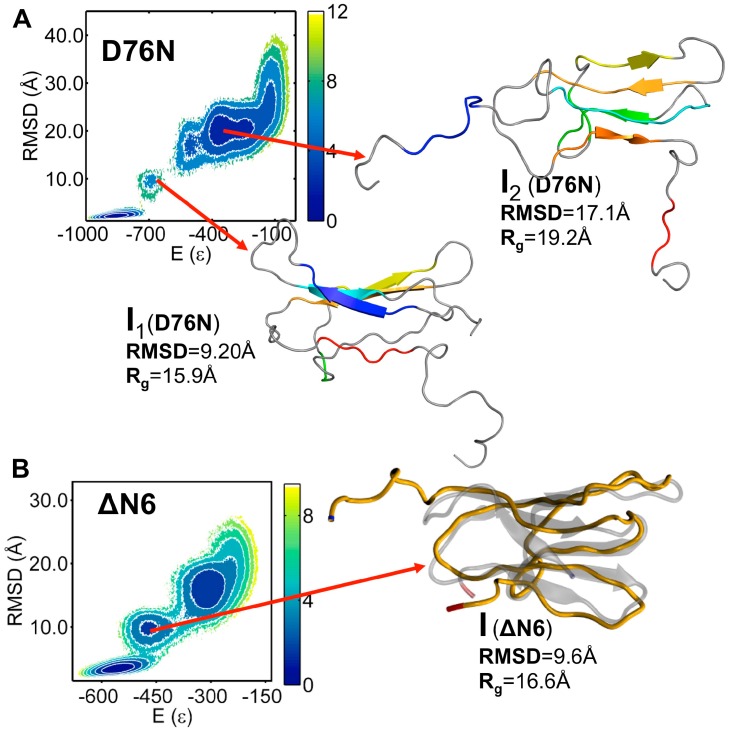
Folding intermediates. (**A**) The two intermediate states populated by the D76N mutant mapped on the folding free energy landscape, which shows a projection of the free energy on the energy and RMSD to the native structure and energy. Both intermediates feature a well-preserved core. While intermediate I_1_ displays an unstructured C-terminal region (C-terminus and G-strand), in intermediate I_2_ both terminal regions are unstructured [33]; (**B**) the intermediate state populated by the ΔN6 variant, in which the core is also preserved but the N-terminal region (N-terminus and A-strand) is unstructured [25].

Previously, we explored the folding transition of the wt b2m, D76N mutant [33] and ΔN6 variant [25] by means of replica-exchange DMD simulations of a full atomistic structure-based Go potential. We found that D76N populates two intermediate states (Figure 3A), which we termed I_1_ and I_2_, featuring a well-preserved core (strands B–F) and showing one (I_1_) or two unstructured termini (I_2_). While I_1_ displayed a 20% increase in SASA relative to the native state, in the case of I_2_ the SASA enhancement reached 53%. In particular, 53% of b2m’s hydrophobic residues became considerably solvent-exposed in the case of I_1_, and 76% in the case of I_2_. While I_2_ was exclusively populated by D76N, I_1_ is conserved across wt and mutant [33]. The ΔN6 variant populated an intermediate state, termed I, which was topologically similar to I_1_ (Figure 3B). In particular, it also featured a well-preserved core and an unstructured and detached terminus [25]. However, in the intermediate I, the labile terminus was the N-terminus instead of the C-terminus. In the intermediate I, about 62% of the hydrophobic residues became considerably more solvent-exposed than in the native state. All intermediates exhibited the trans peptidyl–prolyl bond on the BC-loop, characteristic of the intermediate I_T_.

In order to assess the effect of pH on the structure and dynamics of the intermediate states, we conducted CpHMD simulations where protonable residues were allowed to titrate. The trajectories started from conformations representative of the folding intermediates obtained from structure-based simulations. For both model systems, physiological pH 7.2 was considered. For ΔN6, we also investigated pH 6.2, because this is the pH of the inflamed joints and ΔN6 shows an enhanced in vitro aggregation potential at this pH [29]. For the D76N mutant, we considered a more acidic pH 5.2 because, to the best of our knowledge, the aggregation process of D76N was only assessed under extremely acidic (pH 2.5) [68] and physiological conditions [32,34,68,69].

A detailed analysis of the pH-induced conformational effects at the monomer level was performed in previous studies [25,33], and, here, we focused on its major findings (SI: Tables S1–S3). First, acidic pH induced significant deviations (~16 Å) relative to the native structure of the region comprising strand A and the AB-loop in the intermediate I (ΔN6) (SI Table S1). In the case of I_1_ and I_2_ (D76N), we observed striking deviations of the two terminal regions (up to ~20 Å) both at neutral and acidic pH. This is in line with results reported by Le Marchand [34]. The DE-loop and the EF-loop also exhibited significant deviations from their native positions (up to ~9 Å for I_2_) across the investigated pH values (SI Tables S2 and S3). In the case of intermediate I_1_, we highlighted a comparably striking mobility of the C-terminus (up to ~20 Å), while the N-terminus, DE-loop and EF-loop showed more conservative motions (between 5.4 and 7.6 Å) (SI Table S3).

### 3.2. Dimer Stability under Different pH Conditions

**Figure 4:**
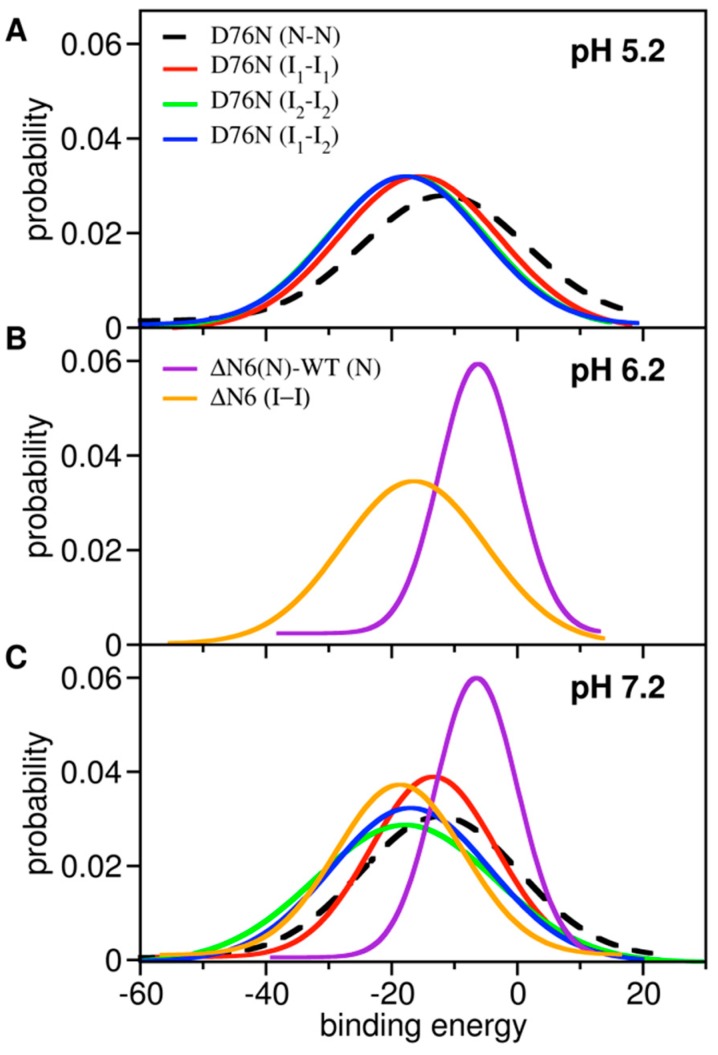
Binding energy. Probability density function for the distribution of binding energy evaluated in each considered ensemble of dimers at acidic pH (**A**), slightly acidic pH (**B**), and neutral pH (**C**).

We started by computing the probability density function (PDF) for the binding energy of dimers composed by monomers (representative of the identified intermediate states) that were extracted from the equilibrated part of CpHMD trajectories at acidic pH (Figure 4A), slightly acidic pH (Figure 4B), and physiological pH (Figure 4C). We also computed the PDF for the binding energy of heterodimers formed by the native state of ΔN6 and the native state of wt b2m, which are the species involved in the ‘prion-like hypothesis’ (Figure 4B). For comparative purposes, we evaluated the PDF for the binding energy of dimers formed by monomers representing the native state of the D76N mutant under different pH conditions. Finally, we evaluated the probability distribution function for the number of intermolecular contacts, which showed that the dimers produced with the deployed docking exhibited a similar degree of compactness, with interfaces reaching ~5K intermolecular atomic contacts (and 250–300 atomic clashes on average) (data not shown).

Under acidic conditions (pH 5.2), the probability density function (PDF) for the binding energy was conserved across the dimers formed by monomers of D76N intermediate states (Figure 3A). The mode (which represents the most likely binding energy within the ensemble of dimers) was approximately the same (E_M_ ~ −18) for dimers of the intermediate I_2_ (i.e., I_2_–I_1_ and I_2_–I_2_ complexes) and slightly higher (E_M_ ~ −16) for homodimers of I_1_.

Interestingly, at pH 7.2, there were noticeable differences in the PDFs. First, the PDF corresponding to I_1_–I_1_ dimers shifted towards higher binding energies. This loss in stability may have resulted from the deprotonation of His84, which in turn was coupled to a smaller mobility and detachment from the core of the C-terminal region. The loss of C-terminal mobility implies that this region became less available to participate in intermolecular interactions. The PDF of I_2_–I_1_ dimers fairly conserved the mode (E_M_ ~ −17), while I_2_–I_2_ dimers got slightly more stable (E_M_ ~ −19) and clearly more stable than I_1_–I_1_ dimers (E_M_ ~ −13) at physiological pH. Furthermore, the tails of the PDF of I_2_–I_2_ dimers extended towards lower energy values with higher probability than at pH 5.2. Taking the binding energy alone as a proxy of dimer stability, one can predict that at physiological pH homodimers of I_2_ will be the most stable. Since dimers must be stable enough to oligomerize further, it is also likely that homo and heterodimers of I_2_ are more prone to aggregate than I_1_ homodimers and are therefore the key species in D76N aggregation. We note, however, that if dimers are too stable, they are likely to remain soluble. Therefore, the most stable dimers, i.e., those pertaining to the tails of the distributions, are not necessarily the ones that will grow into fibrils.

It is interesting to compare the behavior of D76N with ΔN6, which populates the intermediate I. At pH 7.2 and 6.2, I–I dimers had binding energies (E_M_ ~ −19) similar to D76N I_2_–I_2_ dimers, suggesting similar aggregation potential for these two b2m intermediates.

The PDF for the binding energy of heterodimers formed by the native state of ΔN6 and the native state of wt b2m was strikingly shifted towards higher energies with the mode located at E_M_ ~ −6 at both considered pH values, indicating that these dimers are weakly bound. This observation is in line with experimental evidence based on NMR measurements reported by Radford and co-workers [70], who, nonetheless, argued that such a weak binding can still induce conformational changes in the wt b2m protein, originating an amyloidogenic conformation, as well as on ΔN6 itself, while a stronger binding may block the conformational alterations characteristic of a prion mechanism.

### 3.3. Structure of D76N Dimers under Different pH Conditions and Dimerization Hot Spots

**Figure 5:**
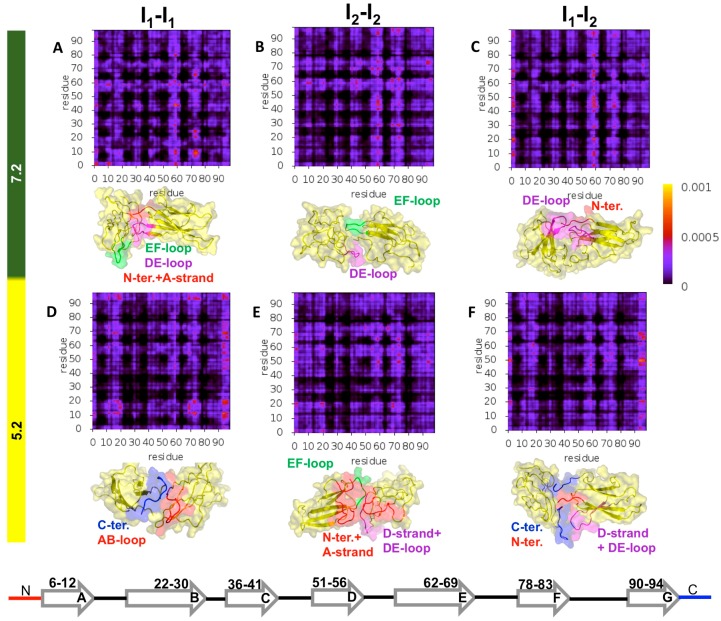
Structural regions involved in D76N dimerization. (**A**–**C**) Probability maps for intermolecular contacts forming at the interface of dimers of the intermediate states populated by the D76N mutant at physiological (**A**–**C**) and acidic pH (**D**–**F**), as well as the three-dimensional representation of representative dimer conformations.

To get insight into the structural features of the D76N dimer’s interface and identify which regions of the monomers are more likely involved in the formation of dimers at acidic and neutral pH, we computed intermolecular probability maps (IPMs) (Figure 5). The IPMs provide the probability of formation of every intermolecular contact established at the dimer’s interface. To properly count contacts within a dimer, it is important to recall that the binding energy contains contributions from three interaction potentials. Therefore, we firstly identified the type of atoms in every possible pair and used the corresponding interaction cut-off to decide if they were within interaction distance (i.e., making a contact). The probability of each intermolecular contact was evaluated by counting the number of times the contact was present in the ensemble of dimers that was used to determine the PDF. A representative dimer conformation (i.e., a conformation with binding energy corresponding to the mode of the PDF) was reported together with the IPM.

**Figure 6:**
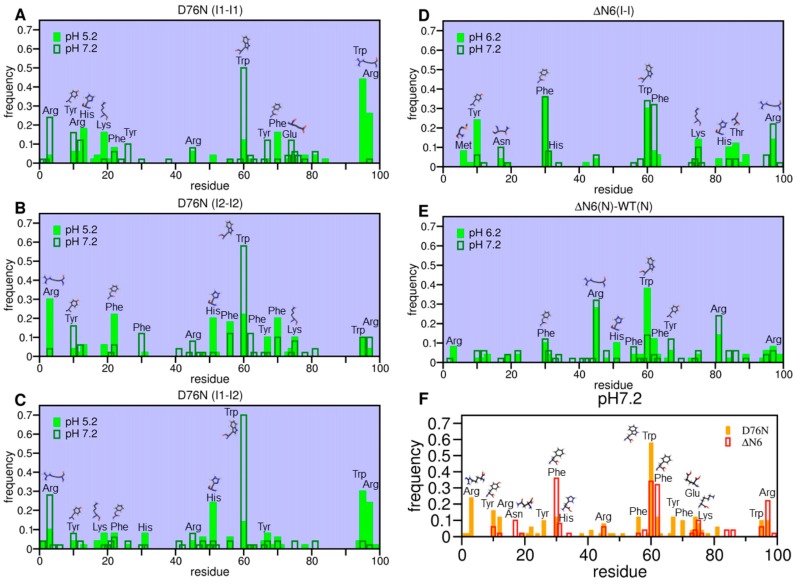
Hot-spot residues. Intermolecular contact probability per residue evaluated in the ensemble of the 50 most frequent intermolecular contacts formed in (**A**) I_1_ homodimers, (**B**) I_2_ homodimers and (**C**) heterodimers of I_1_ and I_2_ intermediate states populated by D76N. (**D**) Homodimers of the intermediate state I populated by the ΔN6 variant and (**E**) heterodimers formed by native conformations of the wt b2m and ΔN6 variant. Additionally shown in (**F**) is the ensemble of hot-spots for D76N and ΔN6 at physiological pH.

To carry out a finer analysis of the dimer interfaces, we identified the so-called dimerization hot-spots (HS), i.e., residues that had a higher tendency to establish intermolecular interactions (Figure 6). Such residues may act as nucleation sites in dimerization (and, by extension, in the aggregation mechanism). Predicting HSs is important, because it represents information that can be tested in vitro (e.g., through mutagenesis). Within the adopted working framework, HSs were identified by computing the probability of intermolecular interaction per residue within the subset of the 50 most frequent intermolecular interactions.

The analysis of the IPMs (Figure 5) indicated that the DE-loop and EF-loop behaved as adhesion zones in the association of all considered intermediate states at pH 7.2 (Figure 5A–C). Their importance, however, was more evident in the association of I_2_–I_2_ dimers at physiological pH. It is possible that the detachment of both the N- and C- terminal regions from the core in the I_2_ intermediate state facilitated (and fosters) the movement of the DE- and EF-loops, in line with observations reported in [34]. Since this mobility enhancement was stronger at pH 7.2 (SI Table S2), the loops will more likely establish intermolecular interactions at physiological conditions. The intermolecular interactions involving these loops became less likely at acidic pH, but their fingerprint was still noticeable in the IPMs (Figure 5D–F)**,** with Phe70 (EF-loop) acting as an HS residue (Figure 6A,B). The leading HS residue at physiological pH was clearly Trp60 (DE-loop), whose role as an interaction hub sharply decreased as the pH was lowered to 5.2. Indeed, under acidic conditions, the dimerization of I_2_ was majorly triggered by Arg3 (N-terminus), followed by two clusters of residues located on the DE-loop and adjoining D-strand (His51, Phe56 and Trp60), and, to a lesser extent, on the EF-loop and adjoining E-strand (Tyr67, Phe70 and Lys75) (Figure 6B). We also pinpointed the participation of Arg3 (N-terminus), Tyr10 and Arg12 (A-strand) in the association pattern of homo and heterodimers, particularly, at physiologic pH.

Under acidic pH, the C-terminus gained relevance as an adhesion zone in the heterodimers and, more strikingly, in homodimers of I_1_ (Figure 5D–F) possibly due to an increased detachment of the C-terminus, which was coupled with increased protonation of His84 (FG-loop) (pK_a_ ~ 5.2). The AB loop also stood out as an important structural element in I_1_–I_1_ dimerization, establishing preferential interactions with the EF loop and AB loop of the other monomer, as well as with the C-terminus. At pH 5.2, I_1_ monomers associated mainly through Trp95 and Arg97 (C-terminus), followed by His13 and Lys19 (both pertaining to the AB-loop) (Figure 6A). The former were also leading HSs in the dimerization of the heterodimers (Figure 6C), where His51 (D-strand) also acted as an HS because of its increased protonation at acidic pH (pKa ~ 6.5).

It is interesting to compare these results with those obtained previously [33], which were based on docking simulations with a cost function that optimized dimers exclusively for shape complementarity. The DE- and EF-loops were already rather important adhesion zones in the dimerization of I_2_ at acidic and physiological pH. However, the C-terminus and the (unstructured) C-terminal region (i.e., C-terminus and G strand) played a strikingly dominant role in I_2_ dimerization at acidic pH that was substantially suppressed when electrostatic and hydrophobic interactions were considered in the cost function. Indeed, the electrostatic interactions involving polar and charged residues of regions such as the N-terminal A-strand were likely accountable for the observed differences. As for the dimerization of I_1_, the AB-loop was already an important driver of monomer association (also at physiological pH), and the participation of the C-terminus in dimerization was significative, in line with the present results.

### 3.4. Structure of ΔN6 Dimers under Different pH Conditions and Dimerization Hot Spots

**Figure 7:**
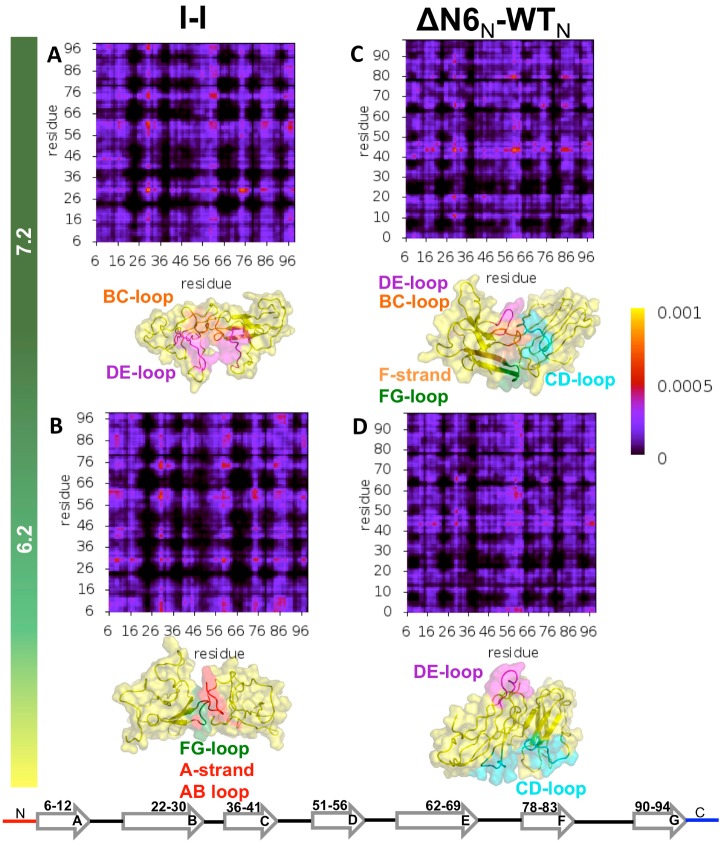
Structural regions involved in ΔN6 dimerization. Probability maps for intermolecular contacts forming at the interface of dimers of the intermediate state I populated by ΔN6, as well as at the interface of dimers resulting from the interaction of the native state of the wt b2m with the native structure of the ΔN6 variant (prion-like hypothesis). Additionally, shown is the three-dimensional representation of representative dimer conformations.

Here we extended the analysis to the ΔN6 variant. We performed a comparative analysis of the dimerization phase of the intermediate I with that triggering a prion-like templating mechanism (Figure 7). According to the latter, the conversion of wt b2m into an aggregation prone conformer was induced by bimolecular collision between the wt protein and the ΔN6 mutant. Therefore, we investigated the structure of dimers formed by the native structure of the wt protein and the native structure of ΔN6.

The analysis of the IPMs revealed that at pH 7.2 the homodimers of the intermediate state I populated by ΔN6 (Figure 7A) associated through the DE-loop and BC-loop, and, to a lesser extent, via the FG loop and the C terminus. When the pH was lowered to 6.2 the N-terminal region (comprising the A-strand and AB-loop) became an important adhesion zone (Figure 7B), in part due to its higher mobility (SI Table S3). The DE-loop conserved its importance, and the interactions involving the FG-loop gained relevance. The major difference between the reported IPMs and those obtained before [25], i.e., with a cost function that optimizes shape complementarity, was a more evident fingerprint for the BC-loop, presumably due to the role of electrostatic interactions established by the His31. It is interesting to note that Trp60 was one of the most significant aggregation HS at physiological and acidic pH (Figure 6D), but its role as an interaction hub in ΔN6 was significantly downgraded when compared with the results for D76N. Phe30 (BC-loop) conserved its role as an HS when the pH was lowered, while His84 and Thr86 (FG loop) saw an enhancement at acidic pH. The HS character of Arg97 (C-terminus) was enhanced at physiological pH, while that of Tyr10 (A-strand) clearly stood out at pH 6.2, presumably because of the increased mobility of the N-terminal region coupled with the increased protonation of His13.

The IPMs for the heterodimers formed by the native state of wt b2m and the native state of ΔN6 revealed an important role of the DE-loop (especially at pH 6.2) (Figure 7D) and of the CD-loop (more pronounced at pH 7.2) (Figure 7C) in the dimerization process. Under physiological pH, the F-strand and the FG-loop also participated, although to a less extent, in protein–protein association (Figure 7C). These results are in line with those reported by Radford and co-workers [70], that claimed the involvement of the DE-loop, BC-loop and FG-loop in the interfaces of the heterodimers of ΔN6 and wt b2m. Trp60 stood out again as an aggregation HS (especially at pH 6.2), and Arg45 (CD-loop) at both physiological and pH 6.2 (Figure 6E). Arg81 (F-strand) was also an important linker at pH 7.2 followed by Arg97 (C terminus), as well as Arg3 (N terminus) at pH 6.2. It is likely that the intermolecular interactions between the positively charged arginine residues may have contributed to destabilize the interfacial region, which is in line with results reported in [70], according to which the high binding energies of these heterodimers arise from an interaction interface that is predominantly electrostatic (instead of hydrophobic) in nature due to a stronger participation of electrostatic interactions (particularly involving residues from the FG-loop).

A comparative analysis of the HS exhibited by the two model systems at pH 7.2 (Figure 6F) allowed us to identify the residues whose importance as potential nucleation sites was conserved across variants. Such residues may be considered relevant to understand b2m aggregation. Our study predicts that Tyr10 (A-strand), Phe30 and His31 (BC-loop), Arg45 (CD-loop), Trp60 and Phe62 (DE-loop), Lys75 (EF-loop), and Trp95 and Arg97 (C-terminus) are key players in b2m dimerization. We propose Tyr10, Lys75, Trp95 and Arg97 as novel dimerization HS because the remaining identified residues have already been proposed to have an important role in b2m dimerization (e.g., His31 in the interface of a ΔN6 nanobody-trapped domain-swapped dimer [30], Arg45 in the interface of DCIM50 covalent homodimers obtained by the substitution of glutamate at position 50 by a cysteine [71], Trp60 in interfaces of b2m dimers [30,39], and Phe62 in the interface of the DimC33 covalent homodimer obtained by the substitution of serine at position 33 by a cysteine [39]).

### 3.5. First Glimpses into the Tetrameritation Phase of D76N

Dynamic light scattering measurements by Vachet and co-workers suggested that in the presence of Cu^2+^ ions and urea the aggregation pathway of wt b2m proceeds exclusively by the formation of even-numbered oligomers (soluble tetramers and hexamers) formed through the addition of dimeric units [40]. In line with this proposal, results from cryo-electron microscopy studies by White et al. [41] indicated that the basic assembly units of the fibril protofilaments are tetramers obtained by a dimer-of-dimers arrangement.

Here, we reported first steps in the study of the tetramerization phase of D76N at physiological pH. The justification for focusing our analysis on the D76N mutant is two-fold: First, it is the most aggregation-prone form of b2m according to studies in vitro [32], and second, the D76N mutant has a clear biological significance [68]. Because the simulations (i.e., protein–protein docking and structure relaxation with molecular dynamics) were significantly time consuming, we restricted our analysis to the most aggregation-prone intermediate state, namely I_2_.

**Figure 8:**
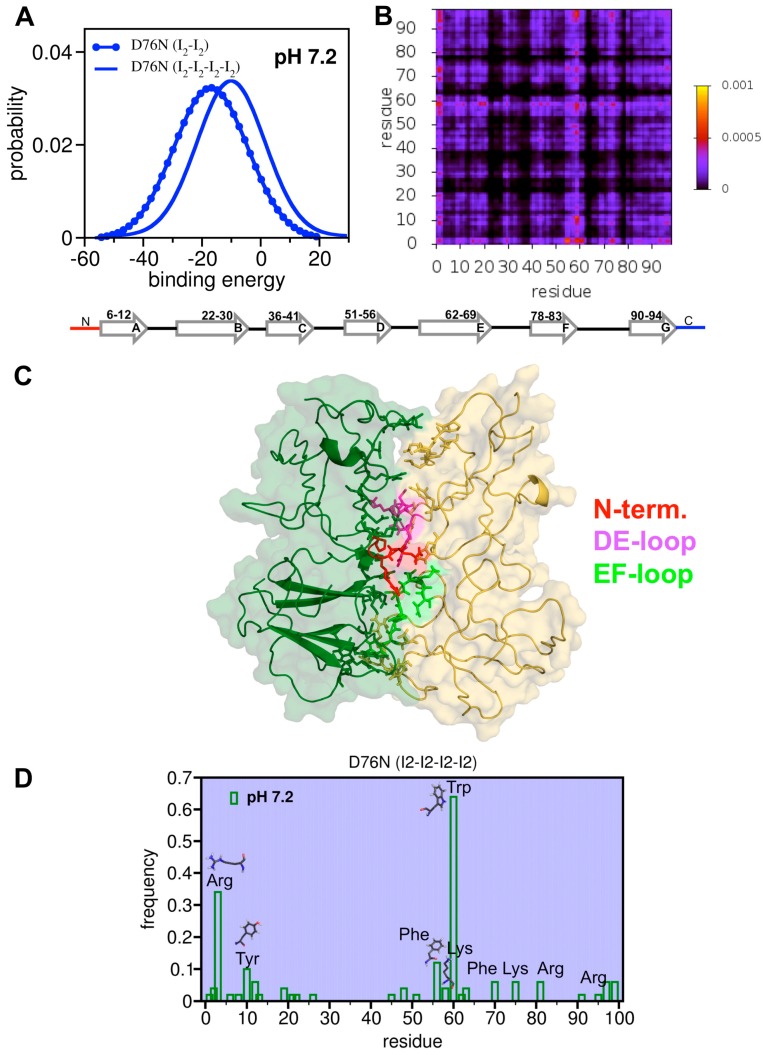
Tetramerization of D76N. (**A**) Probability density function (PDF) for the binding energy of tetramers formed by dimers of I_2_; (**B**) probability map for intermolecular contacts formed at the interface of tetramers; (**C**) representative tetramer conformation in which the dimers are colored green and yellow and the residues that mediate interfacial interactions are represented with sticks; and (**D**) tetramerization hot-spots.

Assuming that the aggregation pathway of the D76N conserves the parity of that of wt b2m, we studied the formation of D76N tetramers by docking dimers of I_2_ at pH 7.2. An ensemble of 1000 tetramers was generated. The PDF for the binding energy indicated that tetramers were significantly less stable (E_M_ ~ −10) than the homodimers of I_2_ (E_M_ ~ −19) (Figure 8A), suggesting that dimers were the most likely dominant species in the initial phase of D76N aggregation. The analysis of the probability map (Figure 8B) for the intermolecular contacts suggested that the N-terminus together with the DE-loop were the most important adhesion zones in the tetramer (Figure 8C). In line with this observation, we found that intermolecular interactions in the tetramer were most likely mediated by 1) Trp60 (DE-loop), 2) Arg3 (N-terminus), 3) Phe56 (D-strand), 4) Tyr 10 (A-strand), and, to a lesser extent, by 5) Lys58 (DE-loop) and 6) Arg97 (C-terminus) (Figure 8D). Trp60 was shown to participate in the interfaces of DCIM20 and DCIM50 tetramers formed by the DCIM50 and DCIM20 covalent homodimers obtained by the substitution of glutamate and serine at position 50 and 20, respectively, by a cysteine [71]. The participation of Lys58, Arg97 and Phe56 in the tetramer’s interface is supported by selective covalent labelling experiments with the wt form in the presence of Cu(II) [72]. Interestingly, while the latter also suggests an important role for the D- and G- strands in the establishment of the tetramer’s interface, they precluded the participation of the N-terminus. However, this observation may result from the fact that the residues located on the N-terminus remained Cu(II) binding sites, and, therefore steric hindrance precluded their participation in the tetramer’s interface.

## 4. Conclusions

Solving the aggregation mechanism of protein beta-2-microglobulin (b2m) is a task of paramount importance given its role as causative agent of dialysis related amyloidosis (DRA), a conformational disorder that affects more than 90% of the individuals undergoing long-term hemodialysis [73].

In vivo, the high affinity of b2m for collagen drives its preferential deposition on the osteoarticular system, which becomes significant when serum concentration attains the micromolar range observed during hemodialysis. It has been proposed that the intense local electric fields due to charge arrays on collagen’s surface energetically destabilize the native structure, which becomes structurally labile and therefore prone to conformationally convert into an amyloid-competent state [3]. Other factors that may influence aggregation in vivo include the interaction of b2m with copper ions (Cu^2+^) [74] (which catalyze the isomerization of the His31–Pro32 bond), medium acidification resulting from inflammation [75], and the presence of molecules like lysophosphatidic acid [76], non-esterified fatty acids [77] and glycosaminoglycans [78].

Unfortunately, the wild-type (wt) form does not aggregate de novo in vitro under physiological conditions, and, over the years, researchers have been exploring engineered or naturally occurring model systems of b2m to gain insight into the fibrillogenesis mechanism of the parent species. The present study focused on the truncated mutant ΔN6, whose biological significance in not clear, and on the single point mutant D76N found in one French family, which aggregates in several visceral organs causing a systemic amyloidosis. While the results reported here help gain insight into the pre-amyloid phase of the parent species in DRA, they do not entail an exclusive role of the truncated species in the actual fibrillogenesis pathway of the full-length wt protein. Aditionally, they do not seek to reduce the latter to the aggregation pathway of the D76N mutant. Indeed, it is likely that aggregation of the full length wt form is strictly dependent on unique environmental conditions occurring in the osteoarticular system of dialysis patients, and, therefore, the latter should be identified and mimicked both in vitro as well as in simulations in order to draw a more accurate picture of wt b2m aggregation in DRA. The goal of the present study is to provide mechanistic insights, hypothesis, and testable theoretical predictions on the early dimerization phase of two model systems (ΔN6 and D76N) that aggregate in vitro under physiological unseeded conditions, which may provide clues and insights into the aggregation mechanism of the wt form.

In particular, we focused our analysis on the self-association process of intermediate states for folding with aggregation potential that were identified in previous simulation studies [25,33] framed on structure-based models for protein folding, i.e., that highlight the topological features of this self-assembly process. A distinctive structural trait of these intermediate states is the existence of one (in the intermediate I of ΔN6, and in the intermediate I_1_ of D76N) or two (in the I_2_ intermediate of D76N) unstructured terminal regions. The importance of unstructured terminal regions in the aggregation mechanism of b2m [11,15,34,74,79,80,81,82,83,84], and in protein aggregation in general has been acknowledged in several studies [85,86,87,88,89,90,91,92].

This study differs from our previous contributions [25,33,44] in one important point: The docking procedure we deployed to explore the dimerization and tetramerizatoin phases of b2m used a cost function that extended beyond packing interactions, i.e., created dimers optimized for shape and energetic complementarity by also considering electrostatic (including hydrogen bonds) interactions), and interactions between hydrophobic atoms.

While the original version of the Monte Carlo ensemble docking (MC-ED) algorithm predicted a direct role (i.e., via the establishment of intermolecular contacts) of the unstructured terminal regions (of all intermediate states) as triggers of aggregation, the method’s version used herein indicates that the unfolding and detachment of the terminal regions from the core may increase the mobility and solvent exposure of other structural elements that now appear as sticky regions, namely the DE-loop (in the dimerization of both b2m variants), the EF-loop (in the dimerization of the D76N mutant), and the BC-loop (in the dimerization of the ΔN6 variant). In particular, the new cost function highlights a more important role for the DE-loop and the EF-loop in the dimerization of the I_2_ intermediate (D76N) at pH 5.2. Overall, the DE-, EF- and BC-loops dominate at physiological pH and the terminal regions at acidic pH. Interestingly, the strikingly dominant role of the C-terminus and adjacent unstructured G strand in the dimerization of I_2_ at acidic pH [33] is substantially suppressed when, besides packing interactions, electrostatic interactions (e.g., those involving polar and charged residues of the N-terminus and A-strand) are also considered. Our results indicate the involvement of residues (1) Arg3, (2) Trp60 and Phe22, (3) His51 and Phe70, (4) Phe56, (5) Tyr67, Lys75, and (6) Trp95 in I_2_ dimerization at pH 5.2, and they also predict the involvement of (1) Arg3, (2) Tyr10, and (3) Arg12 in the homo and heterodimers of I_1_ and I_2_ intermediates at physiological pH.

The results reported here predict a relevant role for the A-strand in the dimerization of ΔN6 under slightly acidic conditions (pH 6.2) and indicate a clear fingerprint for the BC-loop in the dimer’s interfacial region (most likely due to the electrostatic interactions established by His31), which was not detectable with the original version of the docking method. We also analyzed, for the first time, the dimerization interfaces resulting from intermolecular interactions between the native state of ΔN6 and the native state of wt b2m, which would underlie a “prion-like” mechanism for b2m amyloidogenesis. The dimers we obtained are the most unstable of all dimers studied here, featuring relatively high binding energies in agreement with experimental data reported by Radford and co-workers [70]. Our results support the involvement of the DE-loop, BC-loop and FG-loop in the interfaces of the heterodimers of ΔN6 and wild-type b2m, also in line with experimental data [70].

Overall, the results of extensive simulations carried out in the present study are in line with experimental data supporting an essential role for (1) Trp60 (DE-loop), (2) Phe30 (BC-loop), (3) Phe62 (DE-loop), (4) Arg97 (C-terminus), (5) Tyr10 (A-strand), and (6) His31 (BC-loop) in b2m dimerization. Additionally, they predict novel hotspot residues such as (1) Lys75 (EF-loop) and (2) Trp95 (C-terminus). These theoretical predictions could be tested in the context of mutagenesis experiments in which single (or double) point mutations are done to replace the hot-spot residues and the kinetics of aggregation are measured.

Finally, by studying the dimerization of dimers of the intermediate I_2_ populated by the D76N mutant, we obtained first theoretical insights into the tetramerization interface of the mutant. We predict that the N-terminus and DE-loop play an important role in the interface of the tetramer, and we propose that the formation of the latter is mediated by interactions involving (1) Trp60 (DE-loop), (2) Arg3 (N-terminus), (3) Phe56 (D-strand), (4) Tyr 10 (A-strand), and, to a lesser extent, (5) Arg97 (C-terminus), and (6) Lys58 (DE-loop).

## Figures and Tables

**Table 1:** Atomic solvation parameters (cal Å^−2^ mol^−1^) derived by Cummings et al. [64].

*S_C_*	*S_N/O_*	*S_N_^+^*	*S_O_^−^*	*S_S_*
18 ± 2	−7 ± 3	−34 ± 4	−20 ± 8	18 ± 6

## Supplementary Materials

The following are available online at https://www.mdpi.com/2218-273X/9/8/366/s1, Table S1: Cα RMSD of the full length ΔN6 intermediate as well as of specific protein regions measured in relation to the native structure; Table S2: Cα RMSD of the full length intermediate I_2_ as well as of specific protein regions in relation to the native structure; Table S3: Cα RMSD of the full length intermediate I_1_ as well as of specific protein regions measured in relation to the native structure.

## References

[1] Dovidchenko N.V., Leonova E.I., Galzitskaya O.V. (2014). Mechanisms of amyloid fibril formation. Biochem. Biokhimiia.

[2] Chiti F., Dobson C.M. (2006). Protein misfolding, functional amyloid, and human disease. Annu. Rev. Biochem..

[3] Esposito G., Corazza A., Bellotti V., Harris J.R. (2012). Pathological Self-Aggregation of b2-Microglobulin: A Challenge for Protein Biophysics. Protein Aggregation and Fibrillogenesis in Cerebral and Systemic Amyloid Disease.

[4] Chong S.H., Hong J., Lim S., Cho S., Lee J., Ham S. (2015). Structural and Thermodynamic Characteristics of Amyloidogenic Intermediates of beta-2-Microglobulin. Sci. Rep..

[5] Camilloni C., Sala B.M., Sormanni P., Porcari R., Corazza A., De Rosa M., Zanini S., Barbiroli A., Esposito G., Bolognesi M. (2016). Rational design of mutations that change the aggregation rate of a protein while maintaining its native structure and stability. Sci. Rep..

[6] Gumral D., Fogolari F., Corazza A., Viglino P., Giorgetti S., Stoppini M., Bellotti V., Esposito G. (2013). Reduction of conformational mobility and aggregation in W60G beta2-microglobulin: Assessment by 15N NMR relaxation. Magn. Reson. Chem. MRC.

[7] Estacio S.G., Shakhnovich E.I., Faisca P.F. (2013). Assessing the effect of loop mutations in the folding space of beta2-microglobulin with molecular dynamics simulations. Int. J. Mol. Sci..

[8] Narang S.S., Shuaib S., Goyal D., Goyal B. (2018). Assessing the effect of D59P mutation in the DE loop region in amyloid aggregation propensity of beta2-microglobulin: A molecular dynamics simulation study. J. Cell. Biochem..

[9] Natalello A., Relini A., Penco A., Halabelianw L., Bolognesi M., Doglia S.M., Ricagno S. (2015). Wild type beta-2 microglobulin and DE loop mutants display a common fibrillar architecture. PLoS ONE.

[10] Santambrogio C., Ricagno S., Colombo M., Barbiroli A., Bonomi F., Bellotti V., Bolognesi M., Grandori R. (2010). DE-loop mutations affect beta2 microglobulin stability, oligomerization, and the low-pH unfolded form. Protein Sci. A Publ. Protein Soc..

[11] Esposito G., Ricagno S., Corazza A., Rennella E., Gumral D., Mimmi M.C., Betto E., Pucillo C.E., Fogolari F., Viglino P. (2008). The controlling roles of Trp60 and Trp95 in beta2-microglobulin function, folding and amyloid aggregation properties. J. Mol. Biol..

[12] Ricagno S., Colombo M., de Rosa M., Sangiovanni E., Giorgetti S., Raimondi S., Bellotti V., Bolognesi M. (2008). DE loop mutations affect beta2-microglobulin stability and amyloid aggregation. Biochem. Biophys. Res. Commun..

[13] Ricagno S., Raimondi S., Giorgetti S., Bellotti V., Bolognesi M. (2009). Human beta-2 microglobulin W60V mutant structure: Implications for stability and amyloid aggregation. Biochem. Biophys. Res. Commun..

[14] Kihara M., Chatani E., Iwata K., Yamamoto K., Matsuura T., Nakagawa A., Naiki H., Goto Y. (2006). Conformation of amyloid fibrils of beta2-microglobulin probed by tryptophan mutagenesis. J. Biol. Chem..

[15] Calabrese M.F., Eakin C.M., Wang J.M., Miranker A.D. (2008). A regulatable switch mediates self-association in an immunoglobulin fold. Nat. Struct. Mol. Biol..

[16] Blaho D.V., Miranker A.D. (2009). Delineating the conformational elements responsible for Cu(2+)-induced oligomerization of beta-2 microglobulin. Biochemistry.

[17] Rosano C., Zuccotti S., Mangione P., Giorgetti S., Bellotti V., Pettirossi F., Corazza A., Viglino P., Esposito G., Bolognesi M. (2004). beta2-microglobulin H31Y variant 3D structure highlights the protein natural propensity towards intermolecular aggregation. J. Mol. Biol..

[18] Esposito G., Corazza A., Viglino P., Verdone G., Pettirossi F., Fogolari F., Makek A., Giorgetti S., Mangione P., Stoppini M. (2005). Solution structure of beta(2)-microglobulin and insights into fibrillogenesis. Biochim. Biophys. Acta.

[19] Eakin C.M., Berman A.J., Miranker A.D. (2006). A native to amyloidogenic transition regulated by a backbone trigger. Nat. Struct. Mol. Biol..

[20] Ma B., Nussinov R. (2003). Molecular dynamics simulations of the unfolding of beta(2)-microglobulin and its variants. Protein Eng..

[21] Heegaard N.H., Jorgensen T.J., Cheng L., Schou C., Nissen M.H., Trapp O. (2006). Interconverting conformations of variants of the human amyloidogenic protein beta2-microglobulin quantitatively characterized by dynamic capillary electrophoresis and computer simulation. Anal. Chem..

[22] Heegaard N.H., Jorgensen T.J., Rozlosnik N., Corlin D.B., Pedersen J.S., Tempesta A.G., Roepstorff P., Bauer R., Nissen M.H. (2005). Unfolding, aggregation, and seeded amyloid formation of lysine-58-cleaved beta 2-microglobulin. Biochemistry.

[23] Corlin D.B., Sen J.W., Ladefoged S., Lund G.B., Nissen M.H., Heegaard N.H. (2005). Quantification of cleaved beta2-microglobulin in serum from patients undergoing chronic hemodialysis. Clin. Chem..

[24] Heegaard N.H., Roepstorff P., Melberg S.G., Nissen M.H. (2002). Cleaved beta 2-microglobulin partially attains a conformation that has amyloidogenic features. J. Biol. Chem..

[25] Estacio S.G., Krobath H., Vila-Vicosa D., Machuqueiro M., Shakhnovich E.I., Faisca P.F. (2014). A simulated intermediate state for folding and aggregation provides insights into DeltaN6 beta2-microglobulin amyloidogenic behavior. PLoS Comput. Biol..

[26] Fang P.-S., Zhao J.-H., Liu H.-L., Liu K.-T., Chen J.-T., Tsai W.-B., Lin H.-Y., Fang H.-W., Ho Y. (2009). Molecular dynamics simulations to investigate the relationship between the structural stability and amyloidogenesis of the wild-type and N-terminal hexapeptide deletion ΔN6 β2-microglobulin. Mol. Simul..

[27] Hall Z., Schmidt C., Politis A. (2016). Uncovering the Early Assembly Mechanism for Amyloidogenic beta2-Microglobulin Using Cross-linking and Native Mass Spectrometry. J. Biol. Chem..

[28] Esposito G., Michelutti R., Verdone G., Viglino P., Hernandez H., Robinson C.V., Amoresano A., Dal Piaz F., Monti M., Pucci P. (2000). Removal of the N-terminal hexapeptide from human beta2-microglobulin facilitates protein aggregation and fibril formation. Protein Sci. A Publ. Protein Soc..

[29] Eichner T., Kalverda A.P., Thompson G.S., Homans S.W., Radford S.E. (2011). Conformational conversion during amyloid formation at atomic resolution. Mol. Cell.

[30] Domanska K., Vanderhaegen S., Srinivasan V., Pardon E., Dupeux F., Marquez J.A., Giorgetti S., Stoppini M., Wyns L., Bellotti V. (2011). Atomic structure of a nanobody-trapped domain-swapped dimer of an amyloidogenic beta2-microglobulin variant. Proc. Natl. Acad. Sci. USA.

[31] Bellotti V., Gallieni M., Giorgetti S., Brancaccio D. (2001). Dynamic of beta(2)-microglobulin fibril formation and reabsorption: The role of proteolysis. Semin. Dial..

[32] Mangione P.P., Esposito G., Relini A., Raimondi S., Porcari R., Giorgetti S., Corazza A., Fogolari F., Penco A., Goto Y. (2013). Structure, folding dynamics, and amyloidogenesis of D76N beta2-microglobulin: Roles of shear flow, hydrophobic surfaces, and alpha-crystallin. J. Biol. Chem..

[33] Loureiro R.J.S., Vila-Vicosa D., Machuqueiro M., Shakhnovich E.I., Faisca P.F.N. (2017). A tale of two tails: The importance of unstructured termini in the aggregation pathway of beta2-microglobulin. Proteins.

[34] Le Marchand T., de Rosa M., Salvi N., Sala B.M., Andreas L.B., Barbet-Massin E., Sormanni P., Barbiroli A., Porcari R., Sousa Mota C. (2018). Conformational dynamics in crystals reveal the molecular bases for D76N beta-2 microglobulin aggregation propensity. Nat. Commun..

[35] Cohen S.I., Vendruscolo M., Dobson C.M., Knowles T.P. (2012). From macroscopic measurements to microscopic mechanisms of protein aggregation. J. Mol. Biol..

[36] Rennella E., Cutuil T., Schanda P., Ayala I., Gabel F., Forge V., Corazza A., Esposito G., Brutscher B. (2013). Oligomeric states along the folding pathways of beta2-microglobulin: Kinetics, thermodynamics, and structure. J. Mol. Biol..

[37] Eichner T., Radford S.E. (2009). A generic mechanism of beta2-microglobulin amyloid assembly at neutral pH involving a specific proline switch. J. Mol. Biol..

[38] Fabian H., Gast K., Laue M., Misselwitz R., Uchanska-Ziegler B., Ziegler A., Naumann D. (2008). Early stages of misfolding and association of beta2-microglobulin: Insights from infrared spectroscopy and dynamic light scattering. Biochemistry.

[39] Halabelian L., Relini A., Barbiroli A., Penco A., Bolognesi M., Ricagno S. (2015). A covalent homodimer probing early oligomers along amyloid aggregation. Sci. Rep..

[40] Antwi K., Mahar M., Srikanth R., Olbris M.R., Tyson J.F., Vachet R.W. (2008). Cu(II) organizes beta-2-microglobulin oligomers but is released upon amyloid formation. Protein Sci. A Publ. Protein Soc..

[41] White H.E., Hodgkinson J.L., Jahn T.R., Cohen-Krausz S., Gosal W.S., Muller S., Orlova E.V., Radford S.E., Saibil H.R. (2009). Globular tetramers of beta(2)-microglobulin assemble into elaborate amyloid fibrils. J. Mol. Biol..

[42] Estacio S.G., Fernandes C.S., Krobath H., Faisca P.F., Shakhnovich E.I. (2012). Robustness of atomistic Go models in predicting native-like folding intermediates. J. Chem. Phys..

[43] Vila-Vicosa D., Campos S.R., Baptista A.M., Machuqueiro M. (2012). Reversibility of prion misfolding: Insights from constant-pH molecular dynamics simulations. J. Chem. Phys. B.

[44] Krobath H., Estacio S.G., Faisca P.F., Shakhnovich E.I. (2012). Identification of a conserved aggregation-prone intermediate state in the folding pathways of Spc-SH3 amyloidogenic variants. J. Mol. Biol..

[45] Tsuchiya Y., Kinoshita K., Nakamura H. (2006). Analyses of homo-oligomer interfaces of proteins from the complementarity of molecular surface, electrostatic potential and hydrophobicity. Protein Eng. Des. Sel. PEDS.

[46] Jones S., Thornton J.M. (1995). Protein-protein interactions: A review of protein dimer structures. Prog. Biophys. Mol. Biol..

[47] Jones S., Thornton J.M. (1996). Principles of protein-protein interactions. Proc. Natl. Acad. Sci. USA.

[48] Urbanc B., Borreguero J.M., Cruz L., Stanley H.E. (2006). Ab initio discrete molecular dynamics approach to protein folding and aggregation. Methods Enzymol..

[49] Urbanc B., Cruz L., Yun S., Buldyrev S.V., Bitan G., Teplow D.B., Stanley H.E. (2004). In silico study of amyloid beta-protein folding and oligomerization. Proc. Natl. Acad. Sci. USA.

[50] Baker E.N., Hubbard R.E. (1984). Hydrogen bonding in globular proteins. Prog. Biophys. Mol. Biol..

[51] Kortemme T., Morozov A.V., Baker D. (2003). An orientation-dependent hydrogen bonding potential improves prediction of specificity and structure for proteins and protein-protein complexes. J. Mol. Biol..

[52] Ding F., Buldyrev S.V., Dokholyan N.V. (2005). Folding Trp-cage to NMR resolution native structure using a coarse-grained protein model. Biophys. J..

[53] Xu D., Tsai C.J., Nussinov R. (1997). Hydrogen bonds and salt bridges across protein-protein interfaces. Protein Eng..

[54] Seeliger D., de Groot B.L. (2007). Atomic contacts in protein structures. A detailed analysis of atomic radii, packing, and overlaps. Proteins.

[55] Yun S., Urbanc B., Cruz L., Bitan G., Teplow D.B., Stanley H.E. (2007). Role of electrostatic interactions in amyloid beta-protein (A beta) oligomer formation: A discrete molecular dynamics study. Biophys. J..

[56] Sheu S.Y., Yang D.Y., Selzle H.L., Schlag E.W. (2003). Energetics of hydrogen bonds in peptides. Proc. Natl. Acad. Sci. USA.

[57] Brändén C.I., Tooze J. (1999). Introduction to Protein Structure.

[58] Jeffrey G.A. (1997). An Introduction to Hydrogen Bonding.

[59] Teixeira V.H., Cunha C.A., Machuqueiro M., Oliveira A.S., Victor B.L., Soares C.M., Baptista A.M. (2005). On the use of different dielectric constants for computing individual and pairwise terms in poisson-boltzmann studies of protein ionization equilibrium. J. Phys. Chem. B.

[60] Gitlin I., Carbeck J.D., Whitesides G.M. (2006). Why are proteins charged? Networks of charge-charge interactions in proteins measured by charge ladders and capillary electrophoresis. Angew. Chem. Int. Ed. Engl..

[61] Luisi D.L., Snow C.D., Lin J.J., Hendsch Z.S., Tidor B., Raleigh D.P. (2003). Surface salt bridges, double-mutant cycles, and protein stability: An experimental and computational analysis of the interaction of the Asp 23 side chain with the N-terminus of the N-terminal domain of the ribosomal protein l9. Biochemistry.

[62] Biedermann F., Schneider H.J. (2016). Experimental Binding Energies in Supramolecular Complexes. Chem. Rev..

[63] Bickerton G.R., Higueruelo A.P., Blundell T.L. (2011). Comprehensive, atomic-level characterization of structurally characterized protein-protein interactions: The PICCOLO database. BMC Bioinform..

[64] Cummings M.D., Hart T.N., Read R.J. (1995). Atomic solvation parameters in the analysis of protein-protein docking results. Protein Sci. A Publ. Protein Soc..

[65] Fauchere J.-L., Pliska V. (1983). Hydrophobic parameters pi of amino-acid side chains from the partitioning of N-acetyl-amino-acid amides. Eur. J. Med. Chem..

[66] Lesser G.J., Rose G.D. (1990). Hydrophobicity of amino acid subgroups in proteins. Proteins.

[67] Becker J.W., Reeke G.N. (1985). Three-dimensional structure of beta 2-microglobulin. Proc. Natl. Acad. Sci. USA.

[68] Valleix S., Gillmore J.D., Bridoux F., Mangione P.P., Dogan A., Nedelec B., Boimard M., Touchard G., Goujon J.M., Lacombe C. (2012). Hereditary systemic amyloidosis due to Asp76Asn variant beta2-microglobulin. N. Engl. J. Med..

[69] De Rosa M., Barbiroli A., Giorgetti S., Mangione P.P., Bolognesi M., Ricagno S. (2015). Decoding the Structural Bases of D76N ß2-Microglobulin High Amyloidogenicity through Crystallography and Asn-Scan Mutagenesis. PLoS ONE.

[70] Karamanos T.K., Kalverda A.P., Thompson G.S., Radford S.E. (2014). Visualization of transient protein-protein interactions that promote or inhibit amyloid assembly. Mol. Cell.

[71] Colombo M., de Rosa M., Bellotti V., Ricagno S., Bolognesi M. (2012). A recurrent D-strand association interface is observed in beta-2 microglobulin oligomers. FEBS J..

[72] Mendoza V.L., Baron-Rodriguez M.A., Blanco C., Vachet R.W. (2011). Structural insights into the pre-amyloid tetramer of beta-2-microglobulin from covalent labeling and mass spectrometry. Biochemistry.

[73] Jadoul M., Garbar C., Noel H., Sennesael J., Vanholder R., Bernaert P., Rorive G., Hanique G., van Ypersele de Strihou C. (1997). Histological prevalence of beta 2-microglobulin amyloidosis in hemodialysis: A prospective post-mortem study. Kidney Int..

[74] Morgan C.J., Gelfand M., Atreya C., Miranker A.D. (2001). Kidney dialysis-associated amyloidosis: A molecular role for copper in fiber formation. J. Mol. Biol..

[75] Piazza R., Pierno M., Iacopini S., Mangione P., Esposito G., Bellotti V. (2006). Micro-heterogeneity and aggregation in beta2-microglobulin solutions: Effects of temperature, pH, and conformational variant addition. Eur. Biophys. J. EBJ.

[76] Pal-Gabor H., Gombos L., Micsonai A., Kovacs E., Petrik E., Kovacs J., Graf L., Fidy J., Naiki H., Goto Y. (2009). Mechanism of lysophosphatidic acid-induced amyloid fibril formation of beta(2)-microglobulin in vitro under physiological conditions. Biochemistry.

[77] Hasegawa K., Tsutsumi-Yasuhara S., Ookoshi T., Ohhashi Y., Kimura H., Takahashi N., Yoshida H., Miyazaki R., Goto Y., Naiki H. (2008). Growth of beta(2)-microglobulin-related amyloid fibrils by non-esterified fatty acids at a neutral pH. Biochem. J..

[78] Relini A., De Stefano S., Torrassa S., Cavalleri O., Rolandi R., Gliozzi A., Giorgetti S., Raimondi S., Marchese L., Verga L. (2008). Heparin strongly enhances the formation of beta2-microglobulin amyloid fibrils in the presence of type I collagen. J. Biol. Chem..

[79] Motomiya Y., Ando Y., Haraoka K., Sun X., Morita H., Amano I., Uchimura T., Maruyama I. (2005). Studies on unfolded β2-microglobulin at C-terminal in dialysis-related amyloidosis. Kidney Int..

[80] Mukaiyama A., Nakamura T., Makabe K., Maki K., Goto Y., Kuwajima K. (2013). The molten globule of beta(2)-microglobulin accumulated at pH 4 and its role in protein folding. J. Mol. Biol..

[81] Verdone G., Corazza A., Viglino P., Pettirossi F., Giorgetti S., Mangione P., Andreola A., Stoppini M., Bellotti V., Esposito G. (2002). The solution structure of human beta2-microglobulin reveals the prodromes of its amyloid transition. Protein Sci. A Publ. Protein Soc..

[82] McParland V.J., Kad N.M., Kalverda A.P., Brown A., Kirwin-Jones P., Hunter M.G., Sunde M., Radford S.E. (2000). Partially unfolded states of beta(2)-microglobulin and amyloid formation in vitro. Biochemistry.

[83] McParland V.J., Kalverda A.P., Homans S.W., Radford S.E. (2002). Structural properties of an amyloid precursor of beta(2)-microglobulin. Nat. Struct. Biol..

[84] Corazza A., Pettirossi F., Viglino P., Verdone G., Garcia J., Dumy P., Giorgetti S., Mangione P., Raimondi S., Stoppini M. (2004). Properties of some variants of human beta2-microglobulin and amyloidogenesis. J. Biol. Chem..

[85] Mompean M., Chakrabartty A., Buratti E., Laurents D.V. (2016). Electrostatic Repulsion Governs TDP-43 C-terminal Domain Aggregation. PLoS Biol..

[86] Zou Y., Sun Y., Zhu Y., Ma B., Nussinov R., Zhang Q. (2016). Critical Nucleus Structure and Aggregation Mechanism of the C-terminal Fragment of Copper-Zinc Superoxide Dismutase Protein. ACS Chem. Neurosci..

[87] Jarrett J.T., Berger E.P., Lansbury L.P. (1993). The C-terminus of the beta protein is critical in amyloidogenesis. Ann. N. Y. Acad. Sci..

[88] Zheng X., Jia L., Hu B., Sun Y., Zhang Y., Gao X., Deng T., Bao S., Xu L., Zhou J. (2015). The C-terminal amyloidogenic peptide contributes to self-assembly of Avibirnavirus viral protease. Sci. Rep..

[89] Patino M.M., Liu J.J., Glover J.R., Lindquist S. (1996). Support for the prion hypothesis for inheritance of a phenotypic trait in yeast. Science.

[90] Beland M., Roucou X. (2012). The prion protein unstructured N-terminal region is a broad-spectrum molecular sensor with diverse and contrasting potential functions. J. Neurochem..

[91] Smaoui M.R., Mazza-Anthony C., Waldispuhl J. (2016). Investigating Mutations to Reduce Huntingtin Aggregation by Increasing Htt-N-Terminal Stability and Weakening Interactions with PolyQ Domain. Comput. Math. Methods Med..

[92] Baias M., Smith P.E., Shen K., Joachimiak L.A., Zerko S., Kozminski W., Frydman J., Frydman L. (2017). Structure and Dynamics of the Huntingtin Exon-1 N-Terminus: A Solution NMR Perspective. J. Am. Chem. Soc..

