# Supplementary material for: The Early Phase of β2m Aggregation: An Integrative Computational Study Framed on the D76N Mutant and the ΔN6 Variant
Source: Biomolecules. 2019 Aug 14;9(8):366. doi: 10.3390/biom9080366 (PMC6722664; doi:10.3390/biom9080366)
Supplement: Supplementary file 1 [file biomolecules-09-00366-s001.zip › Supplementary_ Materials.docx]

**Supplementary Materials:** The following are available online at www.mdpi.com/xxx/s1, Table S1: Cα RMSD of the full length ΔN6 intermediate as well as of specific protein regions measured in relation to the native structure; Table S2: Cα RMSD of the full length intermediate I_2_ as well as of specific protein regions in relation to the native structure; Table S3: Cα RMSD of the full length intermediate I_1_ as well as of specific protein regions measured in relation to the native structure

**Table S1.** Cα RMSD (Å) of the full-length ΔN6 intermediate I as well as of specific protein regions measured in relation to the native structure.

| **pH** | **Total** | **N21-W94** | **A strand+**  **AB-loop** | **BC-loop** | **DE-loop** | **FG-loop** |
| --- | --- | --- | --- | --- | --- | --- |
| 6.2 | 7.85 ± 0.95 | 5.23 ± 0.57 | 16.08 ± 2.98 | 3.72 ± 1.13 | 7.41 ± 0.83 | 2.44 ± 0.40 |
| 7.2 | 8.08 ± 1.11 | 6.72 ± 1.57 | 13.39 ± 1.52 | 5.95 ± 2.25 | 9.71 ± 2.21 | 3.05 ± 1.07 |

**Table S2.** Cα RMSD (Å) of the full-length D76N intermediate I_2_ as well as of specific protein regions measured in relation to the native structure.

| **pH** | **Total** | **N21-N83** | **A strand+**  **AB-loop** | **BC-loop** | **DE-loop** | **EF-loop** | **C-term.** |
| --- | --- | --- | --- | --- | --- | --- | --- |
| 5.2 | 10.62 ± 0.17 | 3.16 ± 0.05 | 17.99 ± 0.28 | 5.67 ± 0.09 | 8.27 ± 0.13 | 7.23 ± 0.11 | 19.65 ± 0.31 |
| 7.2 | 10.69 ± 0.17 | 3.57 ± 0.06 | 17.90 ± 0.28 | 5.92 ± 0.09 | 9.07 ± 0.14 | 7.69 ± 0.12 | 19.50 ± 0.31 |

**Table S3.** Cα RMSD (Å) of the full-length D76N intermediate I_1_ as well as of specific protein regions measured in relation to the native structure.

| **pH** | **Total** | **N21-N83** | **A strand+**  **AB-loop** | **BC-loop** | **DE-loop** | **EF-loop** | **C-term.** |
| --- | --- | --- | --- | --- | --- | --- | --- |
| 5.2 | 8.15 ± 0.13 | 3.20 ± 0.05 | 7.59 ± 0.12 | 14.17 ± 0.07 | 6.58 ± 0.10 | 7.47 ± 0.12 | 20.45 ± 0.32 |
| 7.2 | 7.67 ± 0.12 | 2.77 ± 0.04 | 6.23± 0.10 | 3.14 ± 0.05 | 5.42 ± 0.09 | 5.90 ± 0.09 | 19.40 ± 0.31 |
